# Rho-Kinase Inhibition Ameliorates Dasatinib-Induced Endothelial Dysfunction and Pulmonary Hypertension

**DOI:** 10.3389/fphys.2018.00537

**Published:** 2018-05-15

**Authors:** Csilla Fazakas, Chandran Nagaraj, Diana Zabini, Attila G. Végh, Leigh M. Marsh, Imola Wilhelm, István A. Krizbai, Horst Olschewski, Andrea Olschewski, Zoltán Bálint

**Affiliations:** ^1^Ludwig Boltzmann Institute for Lung Vascular Research, Graz, Austria; ^2^Institute of Biophysics, Biological Research Centre, Hungarian Academy of Sciences, Szeged, Hungary; ^3^Division of Pulmonology, Department of Internal Medicine, Medical University of Graz, Graz, Austria; ^4^Faculty of Physics, Babeş-Bolyai University, Cluj-Napoca, Romania

**Keywords:** endothelial cells, paracellular permeability, dasatinib, Rho-kinase, pulmonary arterial hypertension, endothelial elasticity

## Abstract

The multi-kinase inhibitor dasatinib is used for treatment of imatinib-resistant chronic myeloid leukemia, but is prone to induce microvascular dysfunction. In lung this can manifest as capillary leakage with pleural effusion, pulmonary edema or even pulmonary arterial hypertension. To understand how dasatinib causes endothelial dysfunction we examined the effects of clinically relevant concentrations of dasatinib on both human pulmonary arterial macro- and microvascular endothelial cells (ECs). The effects of dasatinib was compared to imatinib and nilotinib, two other clinically used BCR/Abl kinase inhibitors that do not inhibit Src. Real three-dimensional morphology and high resolution stiffness mapping revealed softening of both macro- and microvascular ECs upon dasatinib treatment, which was not observed in response to imatinib. In a dose-dependent manner, dasatinib decreased transendothelial electrical resistance/impedance and caused a permeability increase as well as disruption of tight adherens junctions in both cell types. In isolated perfused and ventilated rat lungs, dasatinib increased mean pulmonary arterial pressure, which was accompanied by a gain in lung weight. The Rho-kinase inhibitor Y27632 partly reversed the dasatinib-induced changes *in vitro* and *ex vivo*, presumably by acting downstream of Src. Co-administration of the Rho-kinase inhibitor Y27632 completely blunted the increased pulmonary pressure in response to dasatinib. In conclusion, a dasatinib-induced permeability increase in human pulmonary arterial macro- and microvascular ECs might explain many of the adverse effects of dasatinib in patients. Rho-kinase inhibition might be suitable to ameliorate these effects.

## Introduction

Dasatinib is a second generation BCR/Abl tyrosine kinase inhibitor (TKI) approved for first-and second-line use in patients with chronic myeloid leukemia (CML) and Philadelphia chromosome-positive acute lymphoid leukemia. It is also being evaluated as a therapy for numerous solid cancer types. Besides dasatinib, other TKIs such as imatinib and nilotinib have been approved for the treatment of CML. These TKIs share common targets such as BCR/Abl, platelet derived growth factor receptor (PDGFR) and c-kit. However, dasatinib is also a potent Src tyrosine kinase (SrcTK) inhibitor (Lombardo et al., 2004; Rix et al., 2007).

Due to the differences in the pharmacological profile, each TKI has a unique side-effect profile (Pasvolsky et al., 2015). Dasatinib therapy has been associated with the risk of partially reversible pulmonary arterial hypertension (PAH), with an estimated incidence of 0.45% (Montani et al., 2012, 2013; Orlandi et al., 2012; Sano et al., 2012). To date, more than 100 cases of dasatinib-induced PAH have been submitted for European pharmaceutical vigilance. PAH is a highly morbid and often fatal disease characterized by progressive pulmonary vascular obstruction. PAH affects all layers of the pulmonary arterial wall (Galie et al., 2009; Dayeh et al., 2016). Dasatinib-induced potassium channel inhibition in pulmonary arterial smooth muscle cells may play a key role in the development of PAH, however, adverse effects on endothelial cells (ECs) cannot be excluded (Nagaraj et al., 2013; Olschewski et al., 2014; Guignabert et al., 2016).

Dasatinib therapy may also induce microvascular leakage resulting in pleural effusion, lung and peripheral edema (Han et al., 2013; Latagliata et al., 2013; Dong et al., 2016; Kreutzman et al., 2017). Pleural effusion affects 10–35% of the treated patients, and in 78% the effusion is classified as exudative (with lymphocytic predominance). Dasatinib could affect barrier function through several different mechanisms. Since BCR/Abl has been shown to regulate pulmonary endothelial barrier function (Dudek et al., 2010; Wang et al., 2011), dasatinib may induce dysfunction by the inhibition of BCR/Abl. However, both PAH and pleural effusion are much stronger associated with dasatinib than with nilotinib or imatinib treatment, which suggests that unique actions of dasatinib may cause endothelial leakage and vasoconstriction. Inhibition of potassium channels likely explains pulmonary vasoconstrictive effects but not endothelial leakage.

We investigated the effects of dasatinib on the barrier function of pulmonary micro- and macrovascular ECs, using primary human pulmonary microvascular ECs (HMVEC-L) and pulmonary artery ECs (HPAEC) and employed a number of different cellular and functional readouts. We found that dasatinib but not imatinib or nilotinib caused significant endothelial leakage, associated with Src inhibition and secondary activation of ROCK signaling. We confirmed our *in vitro* results in the isolated perfused rat lung model.

## Materials and Methods

### Cell Culture and Treatments

Human pulmonary microvascular ECs and human pulmonary artery ECs were purchased from Lonza (Allendale, NJ, United States) and were cultured according to the manufacturer’s instructions. In our experiments, we used HMVEC-Ls from four different donors and HPAECs from two different donors. The endothelial-specific media (VascuLife^®^ Basal Medium, Lifeline Cell Technology in the case of HPAEC and EBM-2, Lonza for HMVEC-L) was changed every third day. Cells used in the experiments were between passages five and nine. Confluent cultures were washed with serum-free VascuLife medium, and incubated in the same serum-free medium for 2 h prior to 15 min treatments for western-blot analyses. Transendothelial electrical resistance (TEER), permeability and gene expression measurements were performed in VascuLife medium containing 2% FBS (Sigma). The following concentrations were used for cell treatments: dasatinib (Selleck Chemicals) 1 nM, 10 nM, and 100 nM; imatinib (Selleck Chemicals) 5 μM; nilotinib (Selleck Chemicals) 100 nM; PP2 (Selleck Chemicals) 10 μM; Y-27632 (Tocris) 10 μM and fasudil (Tocris) 10 μM. All chemicals were dissolved in dimethylsulfoxide (DMSO) resulting in 0.1% final concentration during treatments. For control experiments, cells received the same amount of DMSO in medium.

### Transendothelial Electrical Resistance (TEER) Measurements

A computer-controlled device (CellZScope^®^, nanoAnalytics, Muenster, Germany) was used to measure TEER of endothelial monolayers. HMVEC-L cells were cultured until confluence on collagen/fibronectin-coated semipermeable filter inserts (0.4 μm pore size, 0.33 cm^2^, Costar Corning Transwell Clear). Baseline TEER was measured for 1 h before experiments, followed by application of different treatments for up to 24 h.

### Cell Index (CI) Measurements

Cell index (CI) was calculated from real-time impedance data acquired in ACEA’s xCELLigence^®^ real-time cell analysis (RTCA) instrument. HMVEC-L and HPAEC cells were cultured on collagen-coated 96-well E-plates until the CI values reached the plateau. Treatments were applied and changes of endothelial barrier properties were monitored for 12 h.

### Permeability Measurements

Endothelial barrier permeability to 4 kDa FITC-dextran (Sigma) was assessed in phenol red-free VascuLife^®^ Basal Medium (Lifeline Cell Technology) supplemented with 2% FBS (Sigma). After 24 h treatment, medium was removed and 200 μg/ml 4 kDa FITC-dextran containing media was added in the upper compartments. Cultures were incubated at 37°C for 30 min with gentle shaking and samples were collected from the lower compartment. FITC-dextran concentration of the samples was measured using a fluorescent microplate reader (FLUOstar Optima, BMG Labtechnologies, Offenburg, Germany) with an excitation wavelength of 485 nm and an emission wavelength of 520 nm specific for FITC-dextran. Permeability coefficients (*P*) were calculated by the following equation:

$$P=\frac{dQ}{dT\times A\times C_{0}}$$

(*dQ*: transported amount, *dT*: incubation time, *A*: surface of filter, *C*_0_: initial concentration in the luminal compartment).

The values for empty filter inserts (*P*_filter_) were subtracted from the values of the endothelial monolayers (*P*_total_) obtaining the real value for the endothelial monolayer (*P*_e_) using the formula:

$$\frac{1}{P_{e}}=\frac{1}{P_{\text{total}}}-\frac{1}{P_{\text{filter}}}$$

### Immunofluorescence Studies

For immunofluorescence studies, HMVEC-L and HPAEC cells were cultured on collagen/fibronectin-coated filter inserts. Endothelial monolayers were fixed using a mixture of ice-cold ethanol:acetic acid (95:5 v/v) for 10 min and then washed three times for 5 min in PBS. After blocking with 3% bovine serum albumin (BSA, Sigma) for 30 min, inserts were incubated with primary antibodies against VE-cadherin (Cell Signaling Technology) or ZO-1 (Invitrogen), nuclei were counterstained with Hoechst33342 (Sigma). The staining was visualized using Cy3- or Cy2-conjugated secondary antibodies (Jackson Immuno Research) diluted in 1% BSA-containing PBS, and washed three times for 5 min in PBS. Filter inserts were mounted in anti-fading embedding medium (Fluorogel, Electron Microscopy Sciences, Hatfield) and studied using a Nikon Eclipse TE2000U photomicroscope (Tokyo, Japan) connected to a digital camera (Spot RT KE, Diagnostic Instruments, Sterling Heights, MI, United States).

Immunofluorescence images were quantified using the ImageJ software (version 1.51n, NIH). We measured the mean intensity of VE-cadherin immunofluorescence staining using the polygon selection tool to define the cell junctions. In the case of ZO-1 tight junctional protein, we selected the cells by freehand selection tools and then measured the continuity of the immunofluorescence staining.

### Western-Blot Analyses

Cells were lysed in ice-cold lysis radioimmune precipitation buffer (20 mM Tris, 150 mM NaCl, 0.5% Triton X-100, 1% sodium deoxycholate, 0.1% sodium dodecyl sulfate, 1 mM sodium van date, 10 mM NaF, 1 mM EDTA, 1 mM Pefabloc^®^) and incubated on ice for 30 min. Lysates were clarified by centrifugation at 10,000 ×*g* for 10 min at 4°C. Protein concentration was determined with the bicinchoninic acid (BCA) method (Pierce, Rockford, IL, United States). Proteins were electrophoresed with standard denaturing SDS-PAGE procedures and blotted on PVDF (Bio-Rad) or nitrocellulose (Bio-Rad) membranes. Blocking the non-specific binding capacity of the membranes was carried out at room temperature for 30 min in TBS-T (Tris buffered saline with 0.1% Tween 20) containing either 5% casein (non-fat milk powder) or 3% BSA. Membranes were incubated with primary antibodies against total c-Src, pSrc (Y416), or non-phosphorylated (Y416)-Src (Cell Signaling) or β-actin (Sigma). After washing the membranes three times for 10 min in TBS-T, the blots were incubated with the secondary antibodies diluted in TBS-T, then washed again three times for 10 min in TBS-T. The immunoreaction was visualized using Immobilon Western Chemiluminescent HRP Substrate (Millipore, Billerica, MA, United States) on X-ray film (Alga, Mortsel, Belgium).

### Atomic Force Microscopy (AFM) Measurements

All experiments were carried out with an Asylum Research MFP-3D atomic force microscope (Asylum Research, Santa Barbara, CA, United States; driving software IgorPro 6.32A, Wavemetrics), situated on the top of a Zeiss Axiovert 200 type optical microscope. The experiments were performed with overall gold coated silicon nitride rectangular cantilevers holding a V-shaped tip (BL-RC150VB Olympus, Optical Co. Ltd.). The cantilevers have a nominal spring constant of 30 pN/nm, resonant frequency of 25 kHz in air, which drops to 10 kHz in liquid. The spring constant of each of the used cantilevers were determined each time by thermal calibration (Hutter and Bechhoefer, 1993; Higgins et al., 2006; Sader et al., 2012). During measurements cells were kept in Leibovitz’s medium (Gibco, Thermo Scientific) containing 2% FBS at 37°C for maximum 3 h. Images from an area of 60 μm × 60 μm having 256 lines × 256 points were recorded in alternate contact mode using closed loop with a speed of 90 μm/s. Trace and retrace images were recorded and compared for validation, no considerable differences were found. Probing any material with a hard indenter (AFM tip) leads to the theory of indenting an elastic half-space with a stiff object, based on the work of Hertz (1881) and Sneddon (1965) later modified for AFM tips (Mathur et al., 2001). This theory is used for indentation tests, regardless of length scale. Elastic characterization was based on calculating the sample’s elastic modulus from each force curve (Varga et al., 2016).

During recording a single force curve, the tip is brought into contact with the top (indentation below 500 nm) layer of the cells. Force curves were recorded at constant loading speed (8 μm/s) and sampling frequency (2 kHz). Total force distance was kept at 3 μm with maximum load of 300 pN. 32 lines × 32 points maps (force volume) were recorded at each selected area of 60 μm × 60 μm, data collection for one image was approximately 15 min. Values for elasticity were extracted with the microscope’s driving software, implementing the above mentioned models and assumptions.

### *Ex Vivo* Experiments on Isolated Perfused and Ventilated Rat Lungs

All animal care and experimental procedures complied with EU and Austrian regulations and were approved by and reported to the Institutional Animal Care and Use Committee (BMWF, Austria and Medical University of Graz, Graz, Austria). All measures were taken to keep animal suffering to a minimum. Seventeen male Sprague-Dawley rats (Janvier, Saint Berthevin, France), weight 350 ± 112 g (mean ± SD) were used in this study. Rats were sacrificed by an overdose of ketamine (200 mg/kg b.w.) and xylazine (17 mg/kg b.w., exsanguinated, the heart and lung removed enblock and mounted in IPL-2 HSE Harvard Apparatus (March-Hugstetten, Germany). Lungs were perfused through the pulmonary artery at constant flow with 37°C sterile Krebs-Henseleit buffer containing 2% BSA (Sigma A9647), 0.1% glucose, 0.1% Hepes, 24 mM NaHCO_3_ and ventilated with negative pressure in a closed chamber with a tidal volume of ∼12.4 ml/kg b.w., an end-expiratory pressure of -2 cm H_2_O and a respiratory rate of 60 breaths/min. Hyperinflation (-22 to -24 cm H_2_O) was performed at 1-min intervals. Lungs were initially acclimatized with one-way perfusion (∼45′) and then switched to recirculation (total volume 200 ml). After 20′ equilibration period, dasatinib (100 nM) was added to the perfusate (Timepoint 0’). In a subgroup of rats, Y27632 (10 μM) was added to the perfusate 20’ prior to dasatinib. The pH of the perfusate was continuously monitored and adjusted to pH 7.3 with CO_2_. Changes in mean pulmonary arterial pressure and weight (oedema formation) were continuously recorded over a 150-min period. Data are presented as weight/mPAP change compared to the 0’ timepoint. Data were compared using a two-way ANOVA.

### Statistical Analysis

Data analysis was performed using GraphPad Prism software (version 7.0). The statistical tests are presented at each result and given in the figure legends.

## Results

### Dasatinib Alters Barrier Properties of Pulmonary Micro- and Macrovascular Endothelial Cells

**FIGURE 1:**
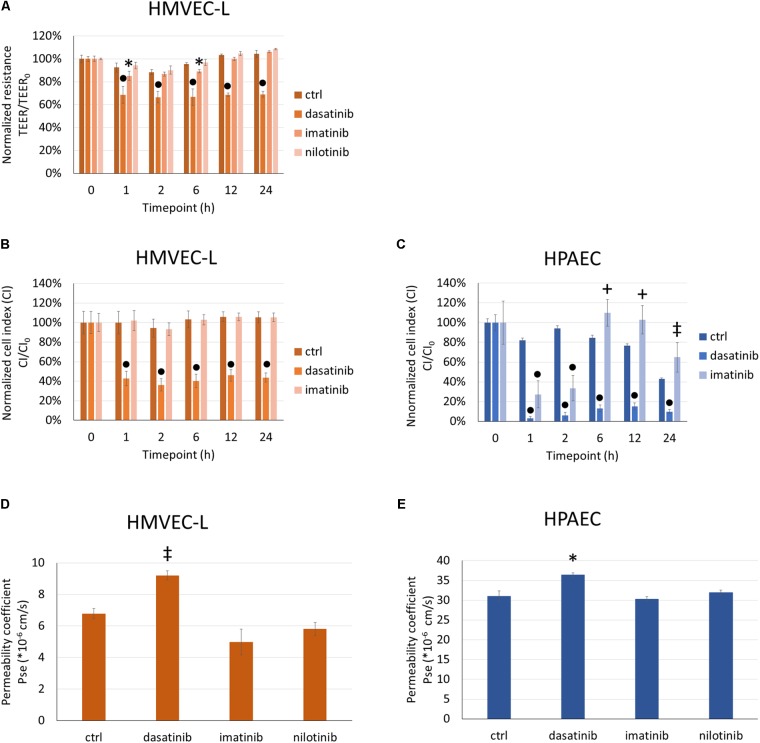
Dasatinib, but not imatinib or nilotinib, alters barrier properties of HMVEC-L and HPAEC cells. HMVEC-L or HPAEC cells were treated with clinically relevant concentrations of dasatinib (100 nM), imatinib (5 μM), nilotinib (100 nM) or vehicle (ctrl: 0.1% DMSO). Cell impedance (transendothelial electrical resistance/TEER or cell index/CI) was followed for 24 h. TEER of HMVEC-L cells was normalized to values before treatments (TEER_0_) **(A)**. CI of HMVEC-L and HPAEC cells were normalized to values before treatments (CI_0_) **(B,C)**. Permeability of HMVEC-L and HPAEC cells to 4 kDa FITC-dextran was measured after 24 h exposure to TKIs **(D,E)**. Data analysis was done by two-way **(A–C)** or one-way **(D,E)** ANOVA with Tukey *post hoc* test. Values are presented as mean ± SD; ^∗^*p* < 0.05, ^‡^*p* < 0.01, ^+^*p* < 0.001, ^∙^*p* < 0.0001 compared to ctrl. Representative results of three (*N* = 3, **A,D**), five (*N* = 5, *B*), four (*N* = 4, **C**) or two (*N* = 2, **E**) independent experiments are shown.

We first evaluated the dasatinib effects on endothelial barrier properties by measuring the electrical impedance of cells exposed to clinically relevant concentrations of dasatinib. For comparison imatinib and nilotinib were also used. Like dasatinib, imatinib, and nilotinib are potent BCR/Abl kinase inhibitors, but less prone to induce PAH or lung edema. In response to 100 nM dasatinib, TEER of microvascular pulmonary endothelial (HMVEC-L) cells dropped significantly within 1 h and this effect lasted up to 24 h (**Figure 1A**). Imatinib at 5 μM had a lower and transient effect on changes in the TEER of HMVEC-L, while nilotinib at 100 nM did not significantly alter the endothelial barrier properties in comparison to control cells (**Figure 1A**).

**FIGURE 2:**
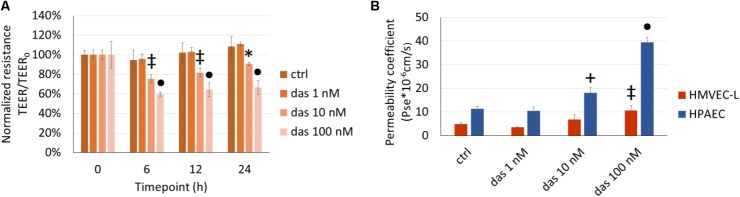
Dasatinib-induced changes in endothelial TEER **(A)** and permeability **(B)** are concentration-dependent. HMVEC-L and HPAEC cells were exposed to dasatinib (das: 1 nM, 10 nM, or 100 nM) or vehicle (ctrl: 0.1% DMSO) for 24 h. Two-way ANOVA test was performed with Tukey *post hoc* multiple comparison test. Values are presented as mean ± SD; ^∗^*p* < 0.05, ^‡^*p* < 0.01, ^+^*p* < 0.001, ^∙^*p* < 0.0001 compared to ctrl. Representative results of three independent experiments (*N* = 3) are shown.

**FIGURE 3:**
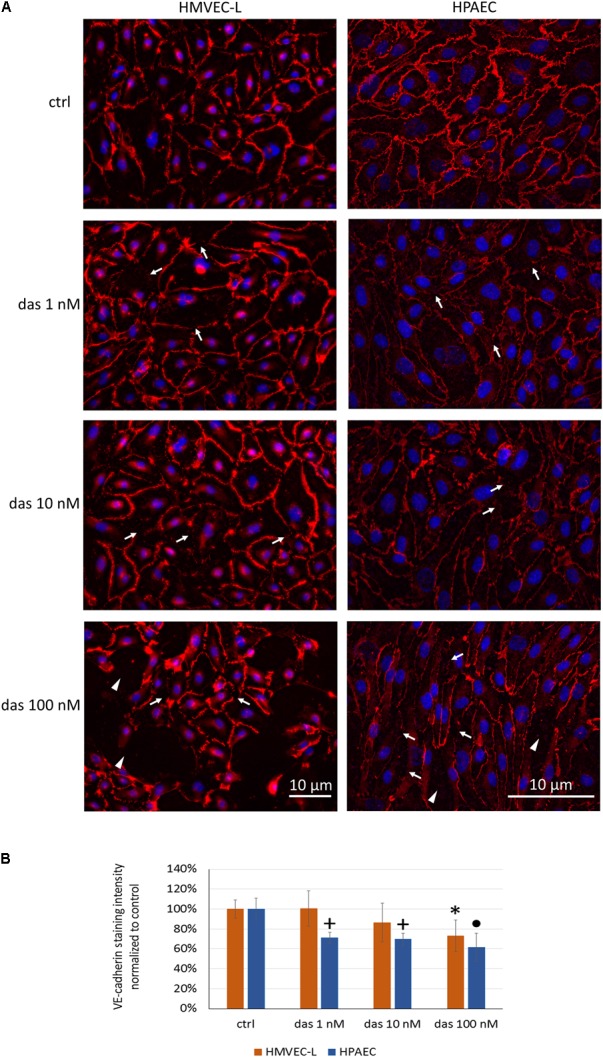
Dasatinib disrupts the junctional complex of HMVEC-L and HPAEC cells in a concentration-dependent manner. HMVEC-L and HPAEC cells were exposed to dasatinib (1 nM, 10 nM, or 100 nM,) or vehicle (ctrl: 0.1% DMSO). Cells were fixed and probed with anti-VE-cadherin antibody (red), nuclei were counterstained with Hoechst 33342 (blue). **(A)** Representative images of two independent experiments are shown. **(B)** Quantification of the immunofluorescence staining intensity. Arrows indicate loss of junctional protein staining, while arrowheads indicate the gaps between cells. Data analysis was done by one-way ANOVA with Tukey *post hoc* test. Values are presented as mean ± SD; ^∗^*p* < 0.05, ^+^*p* < 0.001, ^∙^*p* < 0.0001 compared to ctrl (*N* = 6).

**FIGURE 4:**
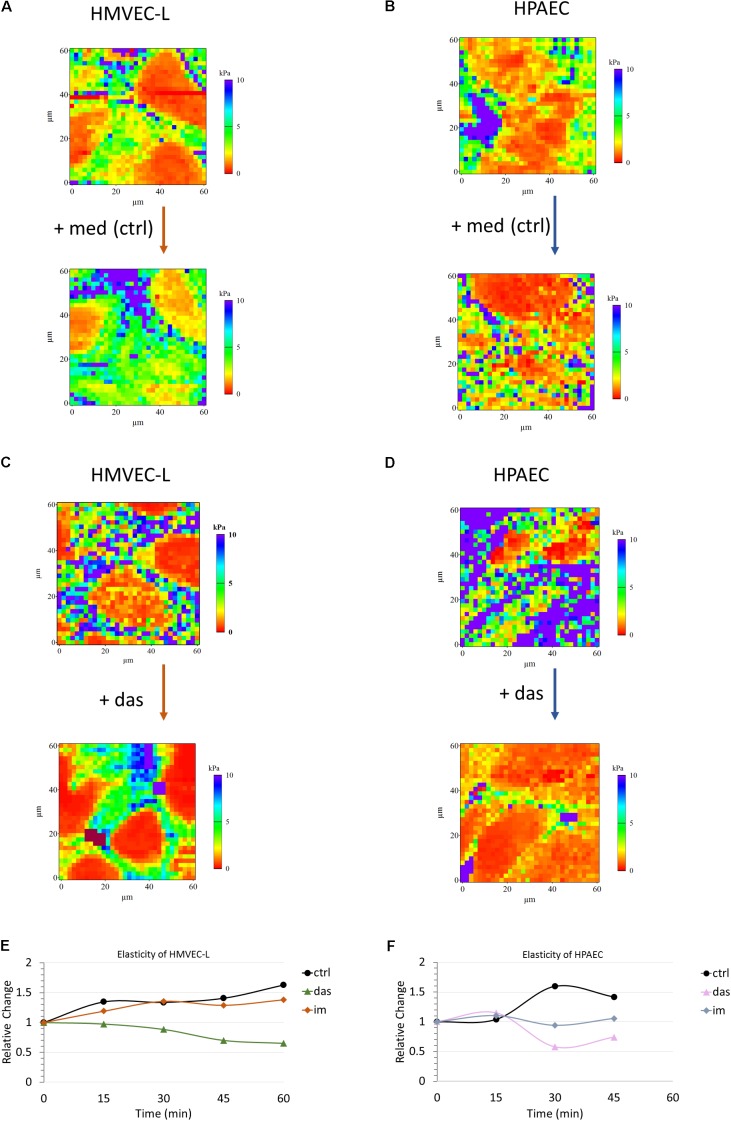
Dasatinib induces changes in elasticity of HMVEC-L and HPAEC cells, as measured by AFM. Pseudo-colored reconstructed elasticity (each point showing the apparent Young’s modulus) maps of HMVEC-L **(A,C)** and HPAEC **(B,D)** cells prior to and 45 min after treatment with vehicle (0.1% DMSO) containing medium (med) as control or 100 nM dasatinib (das), respectively. Charts **(E,F)** represent average values of five parallel sets. Values were normalized to the pre-treatment state of each set. Cells were treated with dasatinib (das, 100 nM), imatinib (im, 5 μM; data not shown) or vehicle (0.1% DMSO) containing medium (ctrl) for both studied cell types.

Since macrovascular ECs do not form such a tight barrier as the microvascular endothelium in the lung, measuring the TEER of cells cultured on semipermeable filter inserts was complemented with measuring the CI of cells cultured on gold electrodes. We applied the same treatments (100 nM dasatinib, 100 nM nilotinib, 5 μM imatinib) in parallel to both micro- (**Figure 1B**) and macrovascular (HPAEC) (**Figure 1C**) ECs and measured in real-time the impedance of the cells reflected by the CI. We observed a rapid decrease of the CI of both HMVEC-L and HPAEC cells after dasatinib application (**Figures 1B,C**). This effect was long-lasting (up to 24 h), whereas imatinib only induced a transient CI decrease in HPAEC cells. After 24 h exposure the permeability of HMVEC-L (**Figure 1D**) and HPAEC (**Figure 1E**) to 4 kDa FITC-dextran was determined. The permeability coefficient reflects the paracellular diffusion of the fluorescent dye, which increased significantly in response to 100 nM dasatinib in both microvascular and macrovascular endothelial monolayers (**Figures 1D,E**). Neither imatinib, nor nilotinib facilitated the transfer of the fluorescent marker from the apical to the basolateral side of the cells.

### The Dasatinib-Induced Pulmonary Endothelial Injury Is Dose-Dependent

The TEER-decreasing effect of dasatinib on HMVEC-L cells proved to be strongly concentration-dependent, with the highest concentration having the most accentuated effect (**Figure 2A**). In accordance with the TEER changes, FITC-dextran permeability assay also showed a concentration- dependent increase in response to dasatinib. These concentration-dependent barrier disrupting effects of dasatinib were observed in both micro- and macrovascular ECs (**Figure 2B**). Dasatinib-induced impedance and permeability changes suggested the involvement of the inter-endothelial junctions. This was confirmed by immunofluorescence staining of the junctional protein VE-cadherin. Treatment of ECs with either 1 nM or 10 nM dasatinib exhibited minimal effects on the distribution of VE-cadherin in HMVEC-L or HPAEC cells. However, the clinically relevant concentration of dasatinib (100 nM) resulted in significant loss of the continuous distribution of VE-cadherin, indicating interendothelial junctional disruption (**Figures 3A,B**). Moreover, we observed formation of inter-endothelial gaps, probably due to changes in the adhesion of the cells to the substrate (**Figure 3A**).

At these concentrations, no apoptotic or cytotoxic effects of dasatinib were detected in the ECs after 24 h of treatment (Supplementary Figures S1A,B). Based on these results, all future experiments were performed with 100 nM of dasatinib.

### Dasatinib Induces Nanomechanical Changes in Pulmonary Endothelial Cells

High resolution three dimensional images were recorded in tapping mode on confluent HMVEC-L monolayers prior to and after 30 min of administration of dasatinib. Dasatinib induced superficial cytoskeletal reorganization and cell morphology alteration which could not be seen in case of the vehicle control (Supplementary Figure S2). Similar results were obtained for HPAEC cells (data not shown). Apparent Young’s modulus was calculated using the modified Hertz model for each recorded curve. Representative reconstructed pseudo-colored elasticity maps recorded prior and after dasatinib treatment or medium change alone are shown for HMVEC-L (**Figures 4A,C**) and HPAEC (**Figures 4B,D**) cells, respectively. In both cases, there was a remarkable increase in elasticity in response to dasatinib as compared to control. As no cell compartment specificity was observed in any case, averages from whole reconstructed maps were calculated for comparison. In order to visualize the dynamics of elasticity change for both cell types, average values from whole maps were calculated and plotted against time. To properly compare the changes induced by different treatments, all values were normalized to initial (pre-treatment) values. **Figures 4E,F** show the time evolution of the relative elasticity change calculated from five parallel measurements for each cell type and treatment. Dasatinib treatment reduced the stiffness of both HMVEC-L (**Figure 4E**) and HPAEC (**Figure 4F**) cells as compared to control. Effect of imatinib treatment on the cells’ elasticity appeared to be similar to control.

### Rho-Kinase (ROCK) Inhibition Attenuates Dasatinib-Induced Endothelial Barrier Dysfunction

**FIGURE 5:**
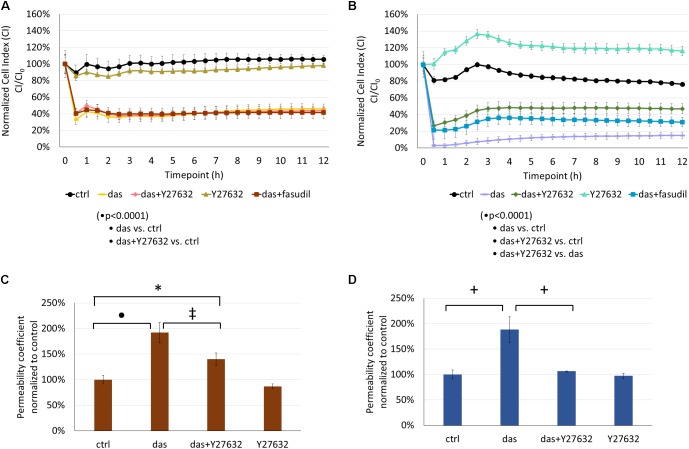
Rho-kinase inhibition partially reverses dasatinib-induced alterations in the barrier properties of HMVEC-L and HPAEC cells. Cells were treated with dasatinib (100 nM), dasatinib and Y27632 (10 μM), Y27632, dasatinib and fasudil (10 μM) or vehicle as control (ctrl: 0.1% DMSO) and CI was assessed **(A,B)**. Permeability changes of HMVEC-L **(C)** and HPAEC **(D)** monolayers was determined after 24 h. Statistical analysis was performed using two-way ANOVA test **(A,B)** or one-way ANOVA **(C,D)** with Tukey *post hoc* multiple comparison; ^∗^*p* < 0.05, ^‡^*p* < 0.01, ^+^*p* < 0.001, ^∙^*p* < 0.0001. Representative images of five (*N* = 5, **A**), four (*N* = 4, **B**) or three (*N* = 3, **C,D**) independent experiments are shown.

ROCK inhibition had no significant effect on the dasatinib-induced impedance drop of HMVEC-L cells (**Figure 5A**). On the other hand, co-treatment with Y27632 or fasudil significantly attenuated the dasatinib-dependent CI decrease of HPAEC cells (**Figure 5B**). The dasatinib-induced permeability increase of HMVEC-L cells to FITC-dextran was moderately decreased in the presence of Y27632 (**Figure 5C**). However, Y27632 significantly reduced hyperpermeability of HPAECs resulting from dasatinib treatment (**Figure 5D**).

**FIGURE 6:**
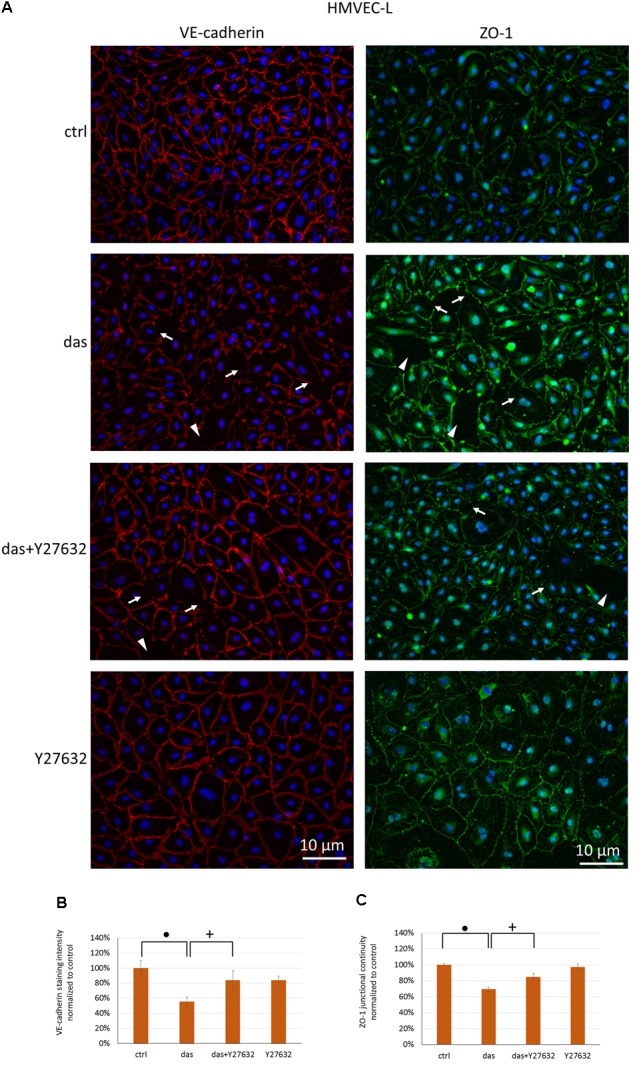
Rho-kinase inhibition partially protects against dasatinib-induced disassembly of endothelial junctions in HMVEC-L cells. Cells were treated with dasatinib (100 nM), dasatinib and Y27632 (10 μM), Y27632 or vehicle (ctrl: 0.1% DMSO) for 1 h. Cells were fixed and probed with anti-VE-cadherin (red) or anti-ZO-1 antibodies (green), nuclei were counterstained with Hoechst 33342 (blue). **(A)** Representative images of three independent experiments are shown. **(B)** Quantification of the immunofluorescence staining intensity and **(C)** staining continuity. Arrows indicate loss of junctional protein staining, while arrowheads indicate the gaps between cells. Data analysis was done by one-way ANOVA with Tukey *post hoc* test. Values are presented as mean ± SD; ^+^*p* < 0.001, ^∙^*p* < 0.0001 compared to ctrl (*N* = 4).

**FIGURE 7:**
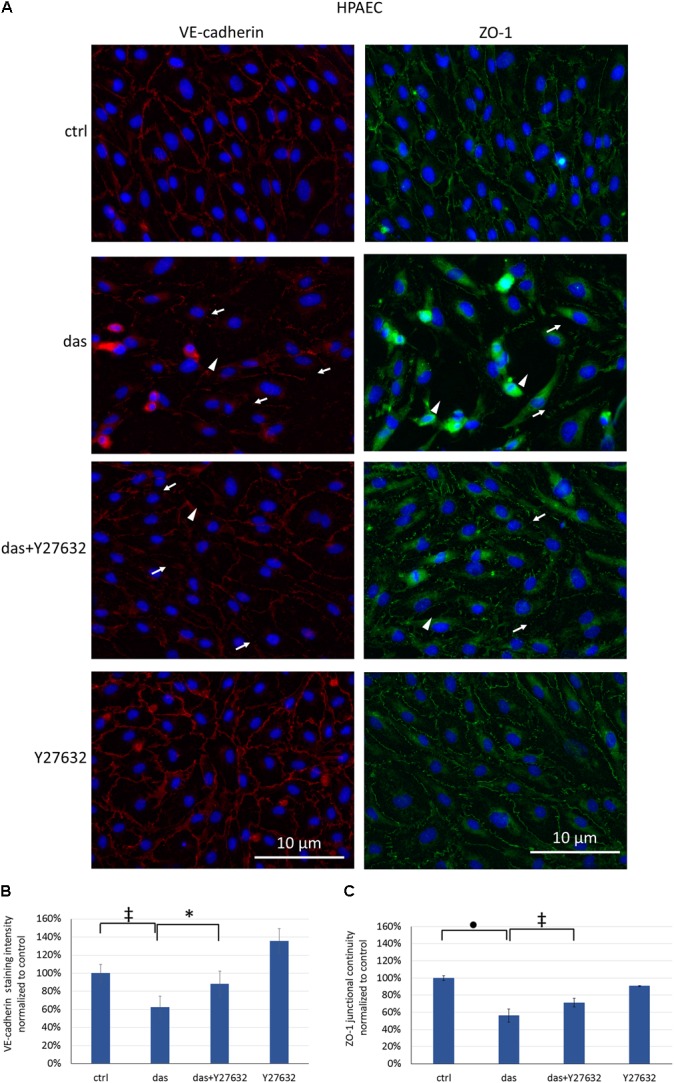
Disruption of endothelial junctions in HPAEC cells is partially ameliorated by simultaneous ROCK inhibition. Cells were treated with dasatinib (100 nM), dasatinib and Y27632 (10 μM), Y27632 or vehicle (ctrl: 0.1% DMSO) for 2 h. Cells were fixed and probed with anti-VE-cadherin (red) or anti-ZO-1 antibodies (green), nuclei were counterstained with Hoechst 33342 (blue). **(A)** Representative images of three independent experiments are shown (*N* = 3). **(B)** Quantification of the immunofluorescence staining intensity **(C)** and staining continuity. Arrows indicate loss of junctional protein staining, while arrowheads indicate the gaps between cells. Data analysis was done by one-way ANOVA with Tukey *post hoc* test. Values are presented as mean ± SD; ^∗^*p* < 0.05, ^‡^*p* < 0.01, ^∙^*p* < 0.0001 compared to ctrl (*N* = 4).

Since ROCK inhibition during dasatinib treatment was beneficial for endothelial barrier properties, we examined the effect of Y27632 on the localization of VE-cadherin and ZO-1 junctional proteins. Y27632 ameliorated the dasatinib-induced loss of endothelial junctional integrity in HMVEC-L cells, as seen both for VE-cadherin and ZO-1 proteins (**Figures 6A–C**). In HPAEC the difference between dasatinib-treated and dasatinib+Y27632-treated cells was even more pronounced, suggesting a particularly beneficial effect of ROCK inhibition (**Figures 7A–C**).

Despite the beneficial effect on the junctions, treatment of ECs with Y27632 did not overcome the cellular stiffness-decreasing effect of dasatinib, as measured with AFM (data not shown).

### Dasatinib-Dependent Src Inhibition Might Contribute to the Disruption of Pulmonary Endothelial Junctional Complexes

Since we observed formation of inter-endothelial gaps in response to dasatinib, we presumed that dasatinib-induced inhibition of Src activity might cause endothelial dysfunction. First, we examined the expression of Src kinases by RT-PCR in HMVEC-L cells. All Src tyrosine kinase mRNA isoforms (c-Src, Yes, Lyn, Fyn, Lck, Frg) were present in these cells, c-Src and Lyn were strongly detected, while Fyn and Yes were detected but weaker. Expression of Lrg and Lyn could nor reliably be detected (Supplementary Figure S1C). HMVEC-L were treated with 100 nM dasatinib for 15 min. Dasatinib markedly reduced Src activation (phosphorylation on Tyr416) whereas the administration of the ROCK inhibitor (Y27632) had no effect on Src phosphorylation (Supplementary Figures S1D,E).

In order to investigate if Src inhibition causes barrier dysfunction in pulmonary ECs, we compared the effects of dasatinib with the Src-specific inhibitor PP2 on TEER and permeability of HMVEC-L monolayers. Similar to dasatinib, PP2 caused a significant decline in the TEER values and an increase in permeability compared to control (Supplementary Figures 3A,B). The permeability increasing effect of PP2 was reversed by Y27632 (Supplementary Figure 3B). In line with the effects of dasatinib, PP2 disrupted the junctional complex of both micro- and macrovascular EC, as shown by decreased junctional staining of ZO-1 and gaps in the monolayers (Supplementary Figure 3C).

### Dasatinib Causes Pulmonary Pressure Increase and Edema Formation in Rat Lungs

**FIGURE 8:**
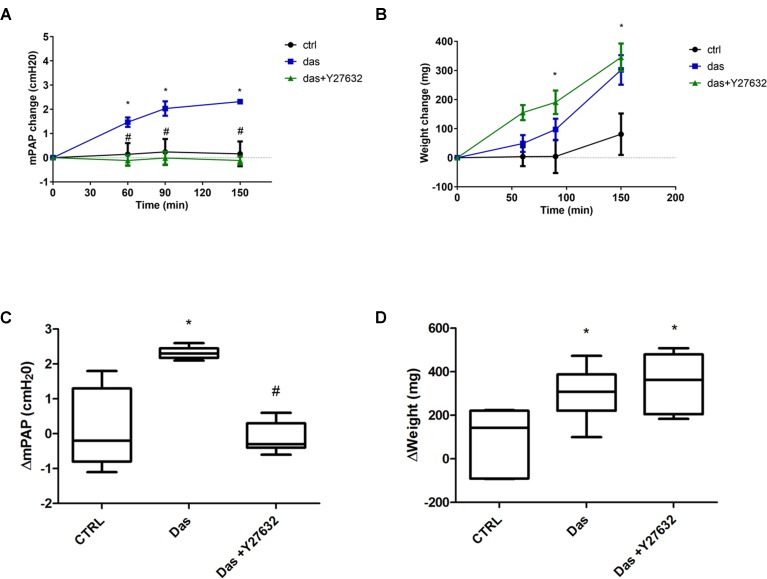
Rho-kinase inhibition ameliorates dasatinib-induced elevation of pulmonary artery pressure, but not lung weight changes/edema formation. Isolated perfused rat lungs were exposed to dasatinib (100 nM), dasatinib and Y27632 (10 μM) or vehicle (CTRL) for 150 min. Changes in mean pulmonary artery pressure (mPAP) **(A)** and lung weight **(B)** were followed in time. **(C,D)** Charts represent lung weight and mPAP change compared to the 0’ timepoint. Data analysis was done by two-way ANOVA, with Bonferroni’s *post hoc* test, five animals/group. Values are presented as mean ± SD; ^∗^*p* < 0.05 as compared to control: CTRL, ^#^*p* < 0.05 as compared to Das.

Our *in vitro* data were complemented with measurements of pulmonary vascular pressure and edema formation in isolated perfused rat lungs. As indicated on **Figures 8A,B**, dasatinib caused an increase in the mean pulmonary arterial pressure, which could be reversed by co-administration of the ROCK inhibitor Y27632. Elevated pulmonary arterial pressure was accompanied by a gain in the lung weight over time (**Figures 8C,D**). However, dasatinib-dependent edema formation was not prevented by Y27632.

## Discussion

The TKIs: imatinib, nilotinib, and dasatinib have been approved for the treatment of CML and primarily target the BCR/Abl kinases and c-kit. Dasatinib, but not imatinib or nilotinib, also potently inhibits the tyrosine kinases of the Src family (c-Src, Yes, Lyn, etc.) (Rix et al., 2007; Weisberg et al., 2007). c-Src has a central role in the regulation of endothelial permeability (Komarova et al., 2017). Dasatinib was shown to effectively inhibit Src in a concentration of 4-9nM (Rix et al., 2007; Shi et al., 2012), while imatinib or nilotinib had no effects up to 10μM (Chislock and Pendergast, 2013) or 4.5 μM (Manley et al., 2010) concentrations.

Off-target effects of dasatinib may be largely responsible for its adverse effects, including PAH, pleural effusion and pulmonary edema. Pleural effusion was characterized by lymphocytosis, suggesting an inflammatory response (Bergeron et al., 2007). PAH could be explained, at least partially, by Src inhibition, causing inactivation of an important two pore domain potassium channel in pulmonary artery smooth muscle cells (PASMCs; Nagaraj et al., 2013), while dasatinib-induced endothelial dysfunction may be independent of Src inhibition. Guignabert et al. (2016) suggested increased ROS production, apoptosis, and elevated expression of endothelial adhesion molecules ICAM-1, VCAM-1, and E-selectin in pulmonary artery ECs as underlying mechanisms. Indeed, increased levels of the adhesion molecules were found in the serum of CML patients (Guignabert et al., 2016). In our study, we induced endothelial dysfunction by exposing human pulmonary macro- and microvascular ECs to clinically relevant concentrations of dasatinib.

After oral administration of 70 mg dasatinib twice daily for CML, the plasma peak concentration (*C*_max_) of dasatinib reaches ∼90 nM (Weisberg et al., 2007). Therefore, we treated pulmonary ECs up to a maximal concentration of 100 nM and assessed the effects on the barrier properties. In accordance with previous data, human pulmonary arterial ECs (HPAECs) have lower relative barrier properties compared to the pulmonary microvascular endothelium (HMVEC-L, Mehta and Malik, 2006; Stevens et al., 2008). Dasatinib treatment increased the permeability of both micro- and macrovascular ECs in a dose-dependent manner and induced a marked decrease in cell impedance. In contrast, the effects of imatinib or nilotinib were minimal. Integrity of the endothelial barrier relies on tight and adherens junctions at cell-cell contacts. The main component of endothelial adherens junctions is VE-cadherin, which is a key regulator of paracellular permeability (Dejana et al., 2009). ZO-1 is a cytoplasmic junctional protein involved in the formation of both tight and adherens junctions (Fanning and Anderson, 2009). Disappearance of VE-cadherin or ZO-1 from intercellular junctions may lead to barrier disruption (Tornavaca et al., 2015). Our results suggest that both VE-cadherin and ZO-1 junctional proteins disappeared from intercellular junctions in micro- and macrovascular endothelial monolayers exposed to dasatinib.

Junctional complexes responsible for the maintenance of barrier properties are connected to the actin cytoskeleton. Cellular responses to agents disrupting intercellular connections or cytoskeletal remodeling can be detected by assessment of the elastic properties of the cells. In order to monitor quick mechanical changes in living cells, atomic force microscope offers an outstanding resolution and precision (Birukova et al., 2009). Using this technique, we followed the changes in elasticity of ECs upon dasatinib or imatinib treatment. We demonstrated that only dasatinib caused softening of both micro- and macrovascular cells. This stiffness decreasing effect may reflect disruption of intercellular junctions and loss of barrier function, as previously shown in brain ECs exposed to hyperosmosis (Balint et al., 2007).

Rho GTPases belong to the main regulators of the actin cytoskeleton. RhoA/ROCK signaling plays an important role in endothelial barrier function through regulation of the actin cytoskeleton. The activated RhoA/ROCK pathway impairs endothelial barrier properties leading to increased paracellular permeability which could explain pleural effusion and edema formation. Several studies suggest that activation of the RhoA/ROCK pathway contributes to PAH (Nakanishi et al., 2016) and that ROCK inhibitors are potential therapeutic agents for PAH (Oka et al., 2008; Fujita et al., 2010). In addition to the regulation of PASMCs, the GTPase RhoA plays primary role in the regulation of endothelial barrier function and permeability. During the development and progression of PAH, endothelial dysfunction is a critical event. Behind the vascular pathology observed in PAH there are changes of signaling mechanisms regulating angiogenesis, endothelial-mesenchymal transition and epigenetic regulation of ECs, which might lead to increased endothelial permeability (Morrell et al., 2009; Good et al., 2015; Ranchoux et al., 2018). The endothelial dysfunction leads to an imbalance of endothelial and smooth muscle cell crosstalk (Morrell et al., 2009; Nogueira-Ferreira et al., 2014). Interestingly, Src blocks the RhoA/ROCK pathway through p190RhoGAP and through phosphorylation of ROCK (Lee et al., 2010). Therefore, it is plausible that the barrier disruptive effect of dasatinib might be due to its inhibitory effect on Src, resulting in ROCK activation. Accordingly, Y27632, a potent ROCK inhibitor ameliorated dasatinib-induced disassembly of VE-cadherin and ZO-1 and the increase in the permeability of endothelial monolayers.

Recently, dasatinib has been shown to induce ROCK-dependent micro- and macrovascular endothelial dysfunction in non-pulmonary ECs. Dasgupta et al. (2017) used human dermal microvascular ECs to show that dasatinib increases endothelial permeability via the Rho-ROCK-MLC (myosin light chain) pathway. Moreover, dasatinib affected tyrosine phosphorylation of p130cas, paxillin, and vinculin, whereas imatinib had no effect on the phosphorylation levels of these proteins. The authors suggested that dasatinib by disrupting the inhibition of RhoA by integrins, promotes activation of RhoA/ROCK pathway, which results in endothelial barrier damage (Dasgupta et al., 2017). Kreutzman et al. (2017) showed that dasatinib causes a profound, reversible and dose-dependent disorganization of HUVEC (human umbilical vein EC) monolayers and that intraperitoneal administration of dasatinib induced intestinal vascular leakage in mice. The molecular mechanisms involved ROCK activation, MLC phosphorylation and activation of non-muscle myosin II. Therefore, involvement of ROCK in dasatinib-induced endothelial dysfunction seems to be important in the whole body, and not only in the lung.

In our study, ROCK inhibition-dependent reduction in dasatinib-induced barrier disruption was more pronounced in pulmonary macrovascular than in pulmonary microvascular ECs, as shown by immunofluorescence staining of junctional proteins, permeability measurements and even more by impedance measurements. Therefore, amelioration of dasatinib complications by ROCK inhibition might be more beneficial in macrovascular pulmonary areas. Accordingly, in isolated perfused and ventilated rat lungs, acute co-administration of the ROCK inhibitor attenuated the dasatinib-induced elevation of pulmonary artery pressure, but had no effects on the weight gain (edema formation) of the lung. In contrast, long-term ROCK inhibition was recently shown to reduce dasatinib-induced Evans blue extravasation in the mouse lung (Dasgupta et al., 2017). Furthermore, in the ROCK1-deficient mouse, dasatinib-induced pulmonary microvascular permeability was reduced to the levels seen in wild type controls. This suggests that long-term ROCK-inhibition might be also effective in amelioration of the pulmonary microvascular dysfunction induced by dasatinib.

According to our data and results from previous studies, the effect of dasatinib on pulmonary ECs may contribute substantially to the pulmonary adverse effects of the drug. Our data are consistent with a scenario in which dasatinib, in pulmonary ECs, by inhibition of src, triggers a barrier disruption that is partly mediated by ROCK, leading to endothelial dysfunction and vascular leakage. The application of a ROCK inhibitor might be beneficial in dasatinib-induced adverse effects involving endothelial dysfunction and pulmonary vasoconstriction.

## Author Contributions

AO, HO, ZB, IK, CF, AV, and IW: conceived and designed the experiments. CF, IW, AV, CN, LM, and DZ: performed the experiments. IK, CF, IW, ZB, LM, AV, AO, DZ, and CN: analyzed the data. IK, CF, ZB, AV, HO, IW, and AO: wrote the paper. All authors approved the final version of the article.

## Conflict of Interest Statement

The authors declare that the research was conducted in the absence of any commercial or financial relationships that could be construed as a potential conflict of interest. The reviewer DHM and handling Editor declared their shared affiliation.
