# Supplementary material for: Rho-Kinase Inhibition Ameliorates Dasatinib-Induced Endothelial Dysfunction and Pulmonary Hypertension
Source: Front Physiol. 2018 May 15;9:537. doi: 10.3389/fphys.2018.00537 (PMC5962749; doi:10.3389/fphys.2018.00537)
Supplement: Supplementary file 1 [file Data_Sheet_1.docx]

**Supplementary material**

**Supplementary methods**

Apoptosis measurements

HMVEC-L cells were plated onto 25 cm^2^ culture flasks in culture medium and were allowed to adhere overnight. The cells were collected by centrifugation after trypsinization and stained with fluorescein-conjugated annexin V and propidium iodide (BD Biosciences, Germany). The apoptosis degree of HMVEC-L cells after 24 hours incubation with the agents was determined as the number of Annexin V positive cells by flow cytometry using a FACS Calibur instrument (BD Biosciences, Germany). DMSO-treated cells were used as vehicle control and 0.05 µM Staurosporine (Cayman Europe) was used as a positive control. Propidium iodide-positive or Annexin V-negative cells were not counted as apoptotic cells.

MTT assay

20.000 HMVEC-L were seeded in 96-well plates and treated for 24 h with 1 nM, 10 nM or 100 nM Dasatinib dissolved in 2% FCS + basal medium or vehicle control (DMSO). As a positive control 1µg/ml Staurosporine was used. After 24 hours, 10 µl MTT (3-(4,5-dimethylthiazol-2-yl)-2,5-diphenyl tetrazolium bromide) was added to each well and cells were incubated for 4 hours at 37°C (MTT was prepared according to manufacture instructions, MTT cell growth assay kit, CT01, Millipore). 100 µl Isopropanol with 0.04 N HCL was then added and absorbence was measured at a wavelength of 570 nm and a reference wavelength of 630 nm within 1 hour.

RT-PCR

Primers and RT-PCR are described previously (Nagaraj et al., 2013). Briefly for reverse transcription (RT) of extracted RNA from the HMVEC-L. The reverse transcription reaction product was directly used in PCR reaction or stored at -20°C. For PCR reactions, 1 μl of 10 pM forward primer, 1 μl of 10 pM reverse primer, 100 ng of cDNA and 12.5 μl of AmpliTaq Gold® 360 Master Mix (Applied Biosystems) were mixed to a final volume of 50 μl with addition of water. The thermal cycler protocol consisted of an initial incubation at 95°C for 5 min, followed by 30 cycles of 95°C for 30 s, 60°C for 30 s and 72°C for 60 s, and a final extension at 72°C for 10 min. The final product was loaded in 1% agarose gel and ethidium bromide was used for the visualization of the product along with the molecular weight marker.
