# Supplementary figures and images for: Rho-Kinase Inhibition Ameliorates Dasatinib-Induced Endothelial Dysfunction and Pulmonary Hypertension
Source: Front Physiol. 2018 May 15;9:537. doi: 10.3389/fphys.2018.00537 (PMC5962749; doi:10.3389/fphys.2018.00537)

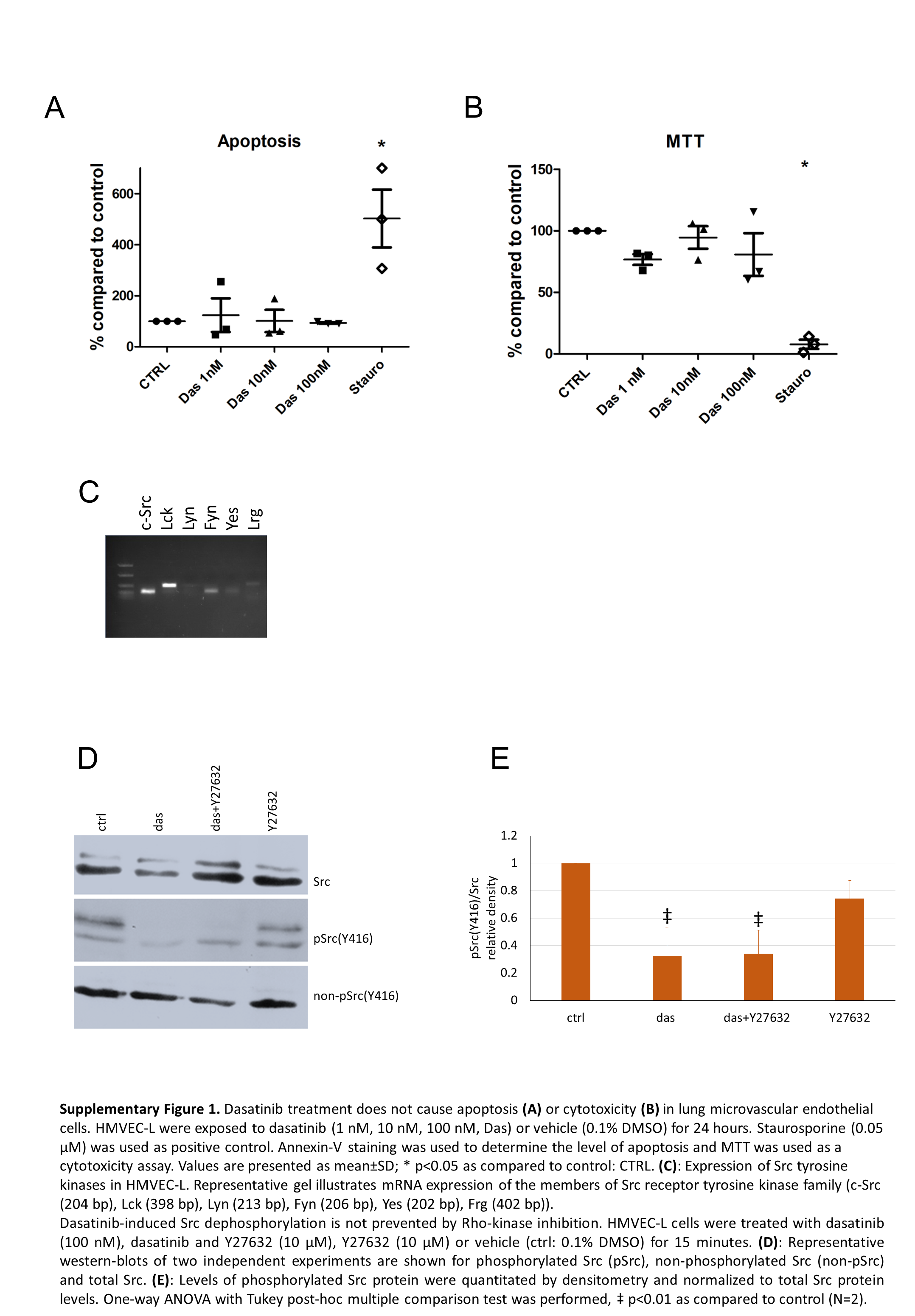

Supplement: Supplementary file 2 [file Image_1.tif]

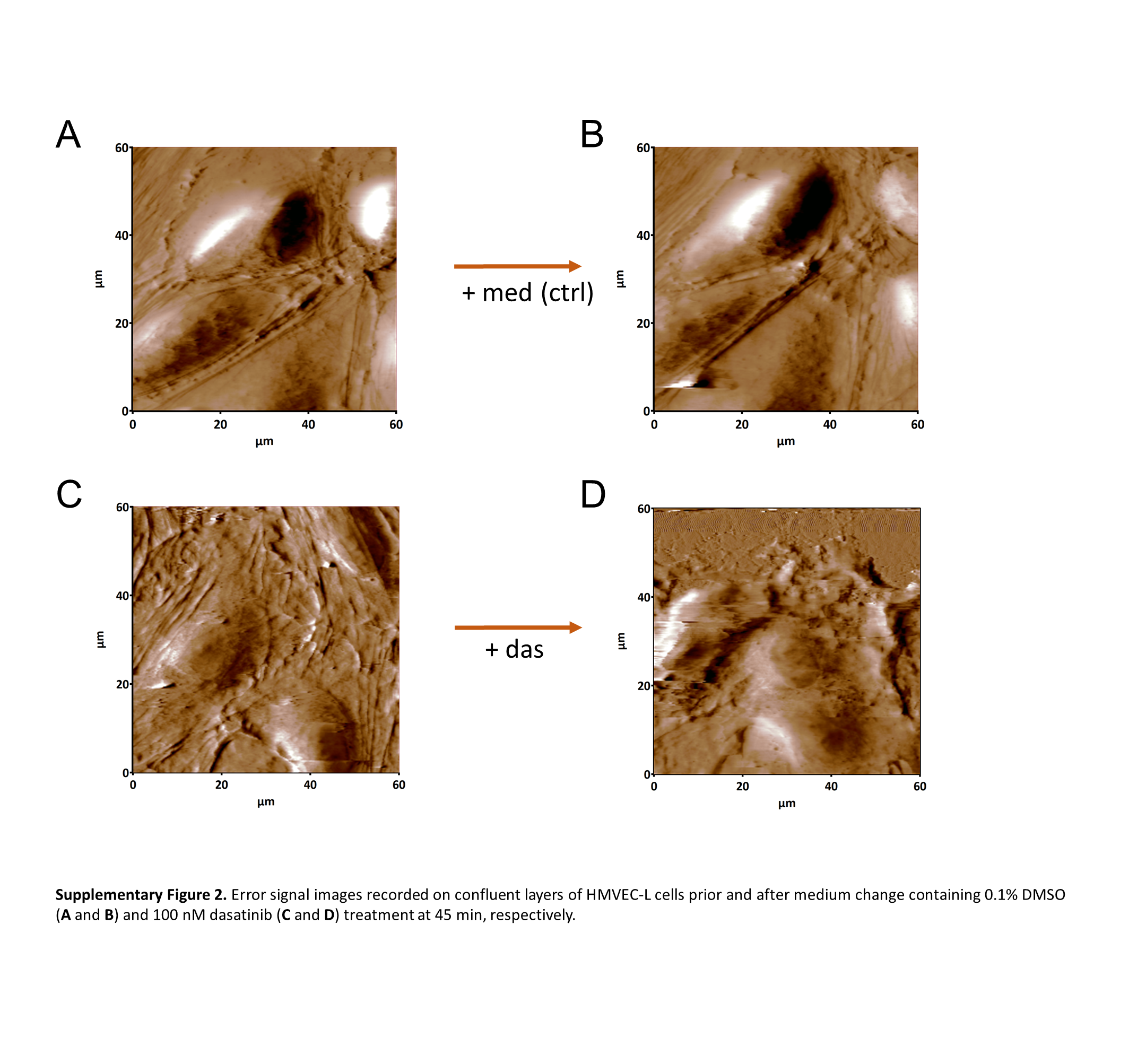

Supplement: Supplementary file 3 [file Image_2.tif]

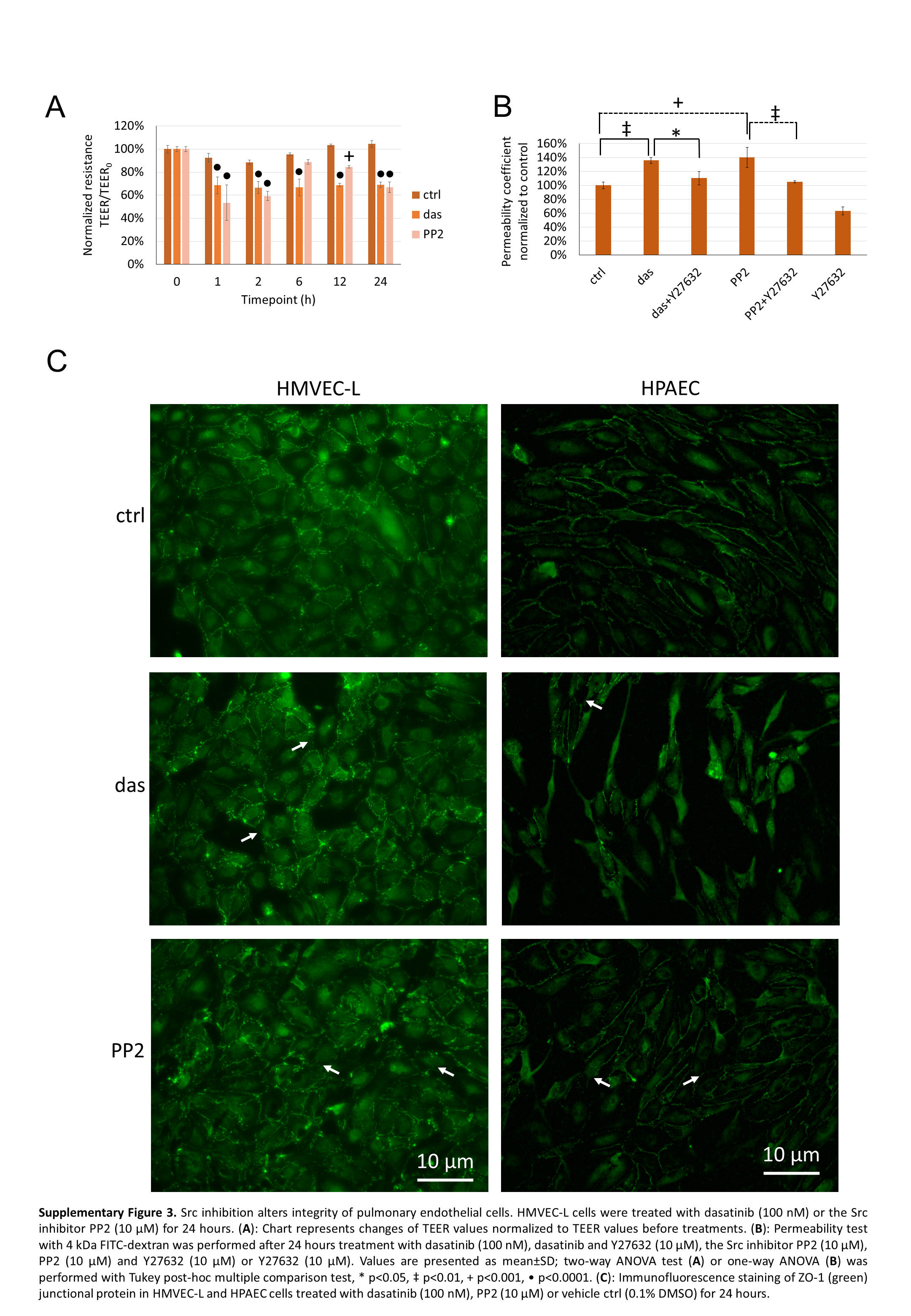

Supplement: Supplementary file 4 [file Image_3.tif]

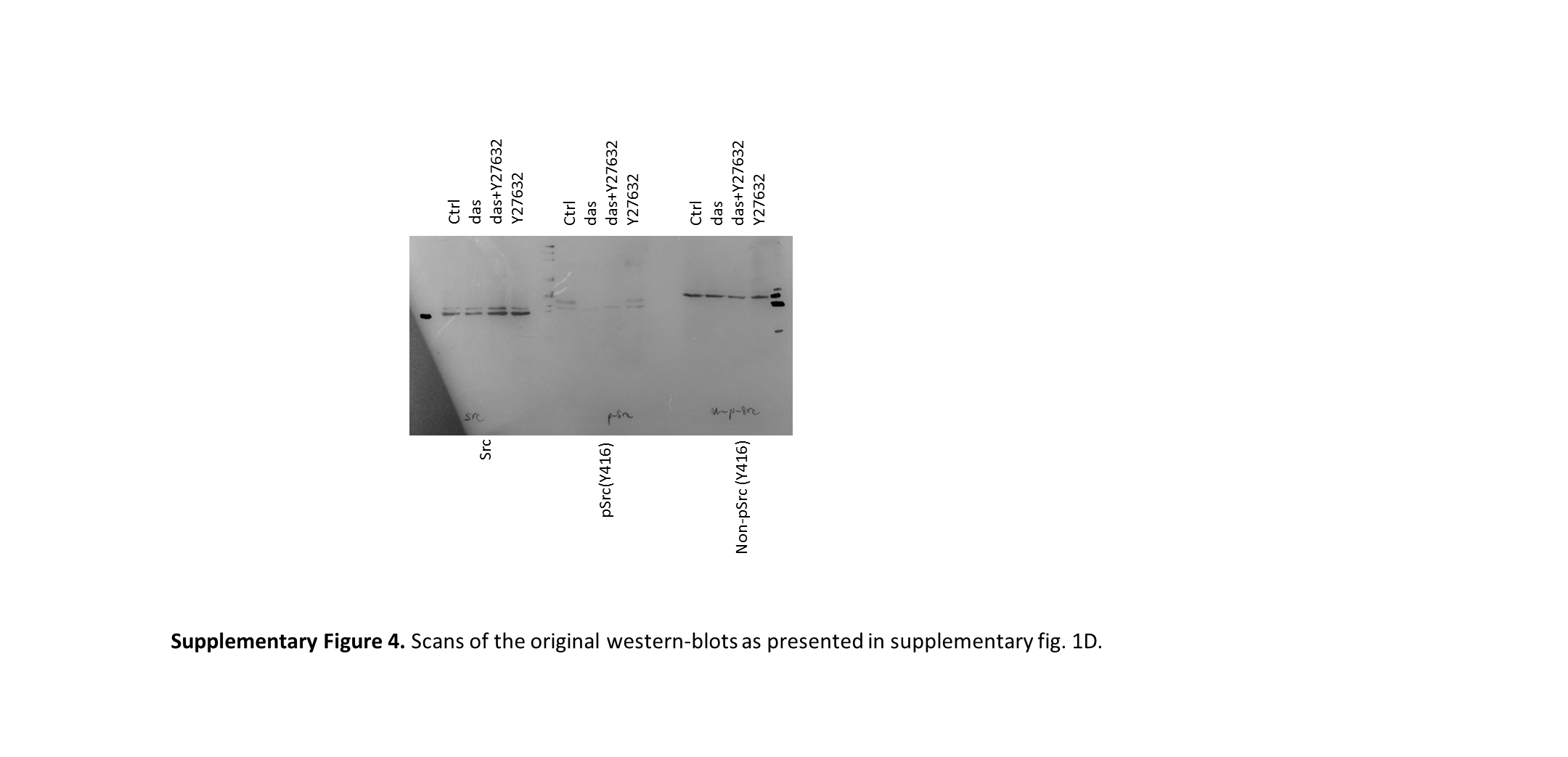

Supplement: Supplementary file 5 [file Image_4.tif]
